# Investigation of Spaceflight Induced Changes to Astronaut Microbiomes

**DOI:** 10.3389/fmicb.2021.659179

**Published:** 2021-06-02

**Authors:** Michael D. Morrison, James B. Thissen, Fathi Karouia, Satish Mehta, Camilla Urbaniak, Kasthuri Venkateswaran, David J. Smith, Crystal Jaing

**Affiliations:** ^1^Physical and Life Sciences Directorate, Lawrence Livermore National Laboratory, Livermore, CA, United States; ^2^KBRwyle, NASA Ames Research Center, Moffett Field, CA, United States; ^3^Department of Pharmaceutical Chemistry, University of California, San Francisco, San Francisco, CA, United States; ^4^Blue Marble Space Institute of Science, Exobiology Branch, NASA Ames Research Center, Moffett Field, CA, United States; ^5^JesTech, NASA Johnson Space Center, Houston, TX, United States; ^6^Biotechnology and Planetary Protection Group, NASA-Jet Propulsion Laboratory, Pasadena, CA, United States; ^7^Space Biosciences Research Branch, NASA Ames Research Center, Moffett Field, CA, United States

**Keywords:** astronaut, international space station, microbiome, metagenomics, spaceflight, microarray

## Abstract

The International Space Station (ISS) is a uniquely enclosed environment that has been continuously occupied for the last two decades. Throughout its operation, protecting the health of the astronauts on-board has been a high priority. The human microbiome plays a significant role in maintaining human health, and disruptions in the microbiome have been linked to various diseases. To evaluate the effects of spaceflight on the human microbiome, body swabs and saliva samples were collected from four ISS astronauts on consecutive expeditions. Astronaut samples were analyzed using shotgun metagenomic sequencing and microarrays to characterize the microbial biodiversity before, during, and after the astronauts’ time onboard the ISS. Samples were evaluated at an individual and population level to identify changes in microbial diversity and abundance. No significant changes in the number or relative abundance of taxa were observed between collection time points when samples from all four astronauts were analyzed together. When the astronauts’ saliva samples were analyzed individually, the saliva samples of some astronauts showed significant changes in the relative abundance of taxa during and after spaceflight. The relative abundance of *Prevotella* in saliva samples increased during two astronauts’ time onboard the ISS while the relative abundance of other commensal taxa such as *Neisseria*, *Rothia*, and *Haemophilus* decreased. The abundance of some antimicrobial resistance genes within the saliva samples also showed significant changes. Most notably, elfamycin resistance gene significantly increased in all four astronauts post-flight and a CfxA6 beta-lactam marker significantly increased during spaceflight but returned to normal levels post-flight. The combination of both shotgun metagenomic sequencing and microarrays showed the benefit of both technologies in monitoring microbes on board the ISS. There were some changes in each astronaut’s microbiome during spaceflight, but these changes were not universal for all four astronauts. Two antimicrobial resistance gene markers did show a significant change in abundance in the saliva samples of all four astronauts across their collection times. These results provide insight for future ISS microbial monitoring studies and targets for antimicrobial resistance screenings.

## Introduction

Decades of research has revealed the important role of microorganisms in maintaining human health. The human microbiome has been shown to be vital in human digestion, metabolism, and immune response. Disruption of the normal commensal microflora has been linked with serious health effects such as the development of periodontal disease (Abusleme et al., 2013; Schwarzberg et al., 2014), cardiovascular disease (Lau et al., 2017), diabetes (Larsen et al., 2010), colorectal cancer (Farhana et al., 2018), and inflammatory bowel disease (Hold et al., 2014; IBDMDB Investigators et al., 2019). While it has not been definitively shown that the dysregulation of the human microbiome directly causes these serious disorders, the onset of colitis (Garrett et al., 2007) and colorectal cancer (Zackular et al., 2013) in healthy animals after fecal transplants supports the idea that the gut microbiome plays a role in the development of these diseases. Therefore, maintaining a healthy “core” microbiome is now widely considered essential for maintaining a healthy lifestyle.

Both government and private space organizations have shown great interest in increasing human access to space with plans of returning to the Moon and landing humans on Mars in the coming decades. One major challenge with renewed long-term spaceflight is the potential risk for human health. Medical treatments during these missions will be limited to the supplies in the on board pharmacy. The onset of in-flight illness is not a new concern for space agencies. Several early Apollo missions had astronauts experience upper respiratory infections, viral gastroenteritis, and urinary tract infections linked to pre-flight exposures (Berry, 1974). Astronauts have experienced reactivation of latent viruses (Mehta et al., 2014, 2017), upper respiratory and urinary tract infections, and skin hypersensitivity (Crucian et al., 2016a) during longer duration missions onboard the Space Shuttle and International Space Station (ISS). Numerous publications have associated the increase in bacterial and viral infections to the dysregulation of the astronaut’s immune system (Kaur et al., 2004; Crucian et al., 2008, 2016a,b). It has been shown that some bacteria are affected by spaceflight and exhibit increased virulence (Wilson et al., 2007, 2008) and antibiotic resistance (Tixador et al., 1985; Moatti et al., 1986; Klaus and Howard, 2006).

NASA has been monitoring astronaut microbiomes throughout the course of human spaceflight. For instance, during the Skylab program, microbial samples were collected from astronauts before, during, and after each mission to investigate how the cultivable microbial populations changed (Johnson and Dietlein, 1977). Air and surface samples from the Apollo and Skylab spacecraft were also collected to monitor cultivable microbial cross-over from the crew and screen for pathogens (Johnston et al., 1975; Johnson and Dietlein, 1977). These studies used traditional culture-based microbiology methods to study the changes of a few selected bacteria and did not provide a comprehensive survey of the overall microbiological changes in the space environment. Additional microbial monitoring experiments have been done recently aboard the Space Shuttle and ISS, coinciding with the implementation of more modern, sequencing-based techniques (Karouia et al., 2017). To better understand the microbiome changes in the ISS, a series of Microbial Tracking studies were conducted as part of the NASA Space Biology research. In the first Microbiome Tracking study, the environmental microbiome of the ISS was characterized to identify the total, viable, and persistent microbes associated with the ISS (Singh et al., 2018; Checinska Sielaff et al., 2019). In the second Microbial Tracking study, astronaut and ISS environmental microbiome were both characterized in order to determine the association between environmental and human microbiome under spaceflight conditions, and if there are any pathogenic microbes that could potentially impact astronauts’ health. Data from the first astronaut and two environmental sampling from the Microbial Tracking -2 project showed that there is microbial exchange between the astronaut microbiome and the ISS environment (Avila-Herrera et al., 2020).

To date, there has been very limited studies on characterization of the astronaut microbiome using metagenomic sequencing and a high-density DNA microarray to detect microbes and pathogens. The NASA twin study was the first metagenomics study to analyze the microbiome changes of one astronaut pre, during and post-flight, though no significant changes were observed (Garrett-Bakelman et al., 2019). This study was limited to gut microbiome only. There were a few reports on astronaut microbiome using 16S rRNA amplicon sequencing. Voorhies et al. (2019) examined astronaut microbiome from gut, skin, nose and tongue, and found that the ISS surface microbiome shared similarities to the skin microbiome. A follow-on 16S rRNA sequencing study of saliva microbiome have correlated changes in the astronaut’s microbiome to viral reactivation (Urbaniak et al., 2020).

In this study, we carried out deep molecular analysis of body microbiome changes from four astronauts pre-, during-, and post-flight. We collected samples from five body sources (i.e., skin, nose, ear, mouth, and saliva) across four astronauts on consecutive spaceflights. For skin samples, five different sections of the skin were sampled including forehead, armpits, forearms (antecubital fossa), and the navel region. Skin and ear have the most contact with the ISS environmental surfaces, therefore they are good sampling locations to study the potential association between human and environmental microbiome. Nose, mouth and saliva samples provide insight on astronauts’ health and physiological conditions. Spaceflight conditions such as radiation and microgravity could also cause dysbiosis of astronaut microbiome and physiology (McQueen and Reilly, 2018; Barratt et al., 2020). Using a combination of metagenomics sequencing, the Livermore Metagenomic Analysis Toolkit (LMAT), and the Microbial Detection Array, we expect to see the composition and relative abundance of the astronaut microbiomes change during spaceflight as compared to pre- and post-flight. We expect that the changes will be different among different astronauts as each human carries a unique set of microbiomes and the effects of spaceflight could influence their microbiome differently. We also expect to see variations between different body site as specific body sites have varied exposures to the ISS environment. A collective survey of these five body parts will give us a broader view of the microbiome changes due to spaceflight conditions.

## Materials and Methods

### Cohort Details and Routine

The cohort was composed of four males between the age of 40–50 years old. The astronaut’s workday is approximately from 6 a.m. to 9:30 p.m. and includes three meals and exercise to maintain muscle tone and fitness. There were no specific restrictions associated with the cohorts’ pre- and post-flight diet that we are aware of. The diet pre- and post-flight were expected to be very different from the in-flight diet as supplies such as fresh fruits and vegetables were limited in-flight. For exercise, the crew follows a daily 2.5-h regimen consisting of treadmill, weightlifting, and biking. Findings have consistently shown astronauts lose 1–5% of pre-flight body mass during spaceflight on both short and long duration flights from the US and Russian space programs, although the situation is improving (Barratt et al., 2020). Finally, the astronauts followed a daily hygiene routine similar to their routine on Earth. However, there is no shower onboard the ISS, so the crew used wetted wipes and rinseless shampoo.

### Sample Collection and Timeline

**FIGURE 1:**
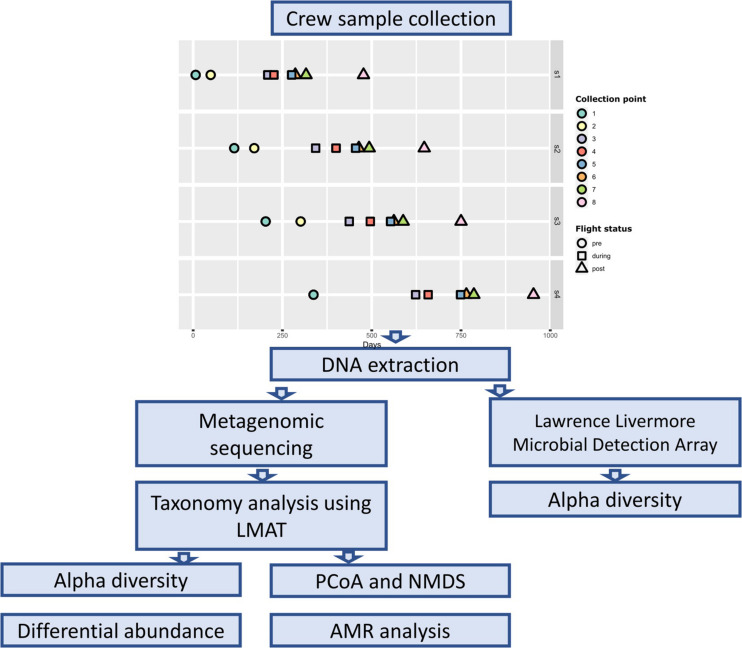
Overall experimental and data analysis workflow and sample collection timeline.

Samples were collected from four astronaut at eight time points (Figure 1). Two pre-flight samples were collected approximately 180 (± 30) and 90 (± 30) days prior to launch (designated as L180 and L90) to establish a baseline (collection points 1 and 2). Three samples were collected throughout the astronaut’s spaceflight, early (1st–2nd months), middle (2nd–4th months) (designated as Flight Day or FD followed by the number of days), and late (10 days before return) (designated as R10, R9, or R5 depending on the number of days before return) (collection points 3–5), to measure spaceflight induced changes. The last three samples were collected 1 day after return, 30 days, and 180 (± 30) days after return (designated as R0, R30, R180) (collection points 6–8). Base line samples collected 90 days prior to launch from Astronaut 4 (s4) were lost; therefore, they were not included in our analysis.

Body swabs and saliva samples were collected as previously described (Avila-Herrera et al., 2020). Briefly, body swabs of the mouth, nasal cavity, forehead, armpits, forearms (antecubital fossa), navel region, and the back of both ears were collected using a polyester swab, EnviroTrans Saline 0.85% Swabs (SRK35 from Hardy Diagnostics, Santa Maria, CA). Swabs were collected first in the morning, with no hygiene 6 h prior to the collection. In addition to these body swabs, saliva samples were collected at each time point. Saliva samples were collected at days 1, 3, 5, and 7 of each time point to capture possible variation across different days. There were a total of 32 saliva samples, 8 pre-flight, 12 during flight, and 12 post-flight. SalivaBio Swabs (Salimetrics, LLC, Carlsbad, CA) were used for saliva collection as described (Mehta et al., 2017). Saliva samples were collected in the morning just after waking up and before eating and drinking, and no exercise 4 h prior to collecting samples. A control body swab was also collected at each time point by waving the swab in the air for 10 s. The samples were frozen at or below −80°C onboard the ISS and during transit back to Earth. Upon return, samples were processed as described previously (Avila-Herrera et al., 2020). DNA extraction was performed using the Maxwell 16 System (Promega, Madison, WI) in accordance with the instructions provided by the manufacturer. Due to the low DNA quantity from the skin swabs, the forehead, armpits, navel, forearms (antecubital fossa), and navel region DNAs from each astronaut were pooled into a single sample labeled as “skin.”

### Metagenomics Sequencing and Taxonomic Classification

Metagenomics sequencing and taxonomic classification were performed as described previously (Avila-Herrera et al., 2020). Briefly, DNA libraries were prepared for sequencing using the Nextera DNA Library Preparation Kit (Illumina, Inc., San Diego, CA). Quality and fragment size were assessed on the Agilent Tapestation 4200 (Agilent Technologies, Santa Clara, CA). Libraries were quantitated using the Qubit fluorimeter (Thermo Fisher Scientific, Waltham, MA) and normalized to equivalent DNA quantities, pooled, and diluted according to the manufacturer’s standard recommendations. Shotgun metagenomic sequencing was performed using an Illumina NextSeq 500 with the NextSeq Series High Output Kit v2 (Illumina Inc., San Diego, CA), using 150 base pair, paired-end reads. Fastq files are generated from the sequencing results using the bcl2fastq software (Illumina). During the fastq generation, sample files are demultiplexed and adapter sequences are trimmed from the reads. The default adapter stringency of 0.9 (90% identity) was used when identifying adapter sequences to trim. Following fastq generation, the ea-utils (Erik Aronesty, 2011) fastq processing utility fastq-mcf was run on all samples with default parameters. The fastq-mcf utility clips poor quality bases (≤Q10) from the ends of reads, removes Ns from read ends, removes reads with the CASAVA “Y” flag, and discards sequences that are too short (<20 bases). Taxonomic classification of the metagenomic reads was accomplished using the Livermore Metagenomics Analysis Tookit [LMAT version 1.2.4b with the “April 4, 2014 LMAT Grand” reference database (lmat-4-14.20mer.db)] (Ames et al., 2013, 2015). Reads mapping to genus *Homo* or below were removed from analysis.

### Microbial Detection Array

All astronaut samples were also analyzed on a microbial detection microarray, the Axiom Microbiome Array (AMA) (Thermo Fisher Scientific, Waltham, MA) to detect the specific microbial content. The AMA is a comprehensive microarray detection system that contains 1.38 million probes to detect 12,513 microbial species representing 135,555 unique target sequences (Thissen et al., 2019). The AMA was adapted from the Lawrence Livermore Microbial Detection Array (LLMDA) which has been used for detection of pathogens, viruses, and bacteria from a variety of clinical and environmental samples. The 96-well array plate version of the AMA was used to run all the astronaut samples on the GeneTitan instrument (Thermo Fisher Scientific, Waltham, MA). A quantity of 20–100 ng of DNA from all astronaut samples was used as input into the AMA. The standard manufacturer’s protocol was followed for all steps of the workflow.

Axiom array controls were included on all array plates that were run. Each plate included one positive Axiom Reference Genomic DNA 103 (Ref103) and one no template negative control (NTC). Controls were processed along with the samples and are used for generating assay QC and array QC metrics following standard manufacturer protocols. Briefly, the Axiom Microbiome Array includes human specific probes that are used to generate the QC metric Dish QC (DQC) for the Ref103 control. DQC is calculated based on the intensities of the probe sequences in non-polymorphic human genome locations and is a measure of the distribution of fluorescent intensity values found on the individual bases. A DQC value of less than 0.82 in the Ref103 control indicates a probable issue with the processing of a plate. All Ref103 controls in this study had DQC values that exceeded 0.82. The NTC was run to ensure that no contaminating microbial organisms were found in the microarray reagents.

Data analysis was carried out using the Axiom Microarray Detection Analysis Software (MiDAS) (Thermo Fisher Scientific, Waltham, MA). The Axiom MiDAS analysis software requires that at least 20% of target-specific probes have a signal intensity above the 99th percentile of the control probes for a positive result. The prevalence of opportunistic pathogens was calculated for each body location pre-, during-, and post-flight. Significance testing of prevalence between flight statuses was performed using the prop.test function available in the stats package in R, and all *P*-values were adjusted using the Benjamini-Hochberg method.

### Alpha Diversity

The alpha diversity of each sample was calculated using the renyi and estimateR functions available through the vegan package (v.2.5-5) (Oksanen et al., 2019). For the LMAT results, the Renyi entropy of each sample was calculated for orders 0, 1, and 2, or Hill numbers (Hill, 1973), representing the sample’s richness, Shannon index, and Simpson index, respectively. To identify changes in the alpha diversity indices throughout the experiment, we analyzed the astronaut samples as a whole and on an individual level. Samples collected at corresponding time-points from all four astronauts were grouped together and statistical testing was performed between the time-points. Alpha diversity changes from individual astronauts were determined by grouping samples by flight status (i.e., pre-, during-, or post-flight). Significance testing was performed using the Kruskal-Wallis test available in the R stats package (R Core Team, 2020) with a significance cut off *P* < 0.05.

### Ecological Distance and Ordination

The similarity between astronaut samples was measured using the distance and visualized using the ordinate functions from the phyloseq package (v.1.24.3) (McMurdie and Holmes, 2013). Distances between samples were calculated using the Euclidean and Jaccard distance and visualized by classical (PCoA) and non-metric multidimensional scaling (NMDS), respectively. The Jaccard distance was used to measure the difference in the taxa presence/absence profiles between the individual samples, and the Euclidean distance was used to measure the differences in each taxon’s relative abundance between samples. Prior to calculating the Euclidean distance, the read counts for each sample were transformed into Euclidean space via the center-log ratio (clr) transformation using the ALDEx2 (Fernandes et al., 2013) package in R.

A permutational multivariate analysis of variance (PERMANOVA) (Anderson, 2001, 2017) was conducted on the Jaccard and Euclidean distances to test for differences in the centroid and distribution of sample groups. PERMANOVA tests were performed using the adonis2 function from the vegan package (v.2.5-5) (Oksanen et al., 2019). PERMANOVA testing was performed on each body location separately, and samples were grouped by either astronaut or flight status. Astronauts were also analyzed individually using the saliva samples, and the samples were grouped according to their flight status (i.e., pre-, during-, or post-flight).

### Differential Abundance Analysis

Differential abundance analysis was performed on the astronaut saliva samples due to the increased number of samples at each collection point (*n* = 4). Read counts were transformed using the clr method (Aitchison, 1982) prior to testing for differentially abundant taxa. All differential analysis testing was performed within each astronaut individually. Differential abundance was determined using the aldex.kw function from the ALDEx2 package (v.1.16.0) (Fernandes et al., 2013). *P*-values were adjusted for multiple testing bias using the Benjamini and Hochberg method (Benjamini and Hochberg, 1995). All taxon with an adjusted *P*-value < 0.05 were considered differentially abundant.

### Antimicrobial Resistance Marker Analysis

Identification of antimicrobial resistance (AMR) gene markers was accomplished using the ShortBRED pipeline (Kaminski et al., 2015) available at https://github.com/biobakery/shortbred. The pre-built “ShortBRED_CARD_2017_markers” dataset available on the ShortBRED bitbucket website was used for the AMR marker dataset throughout this analysis. All astronaut saliva samples were input into ShortBRED for AMR marker quantification. Significant changes in AMR counts between flight pre-, during-, and post-flight samples were determined using the Kruskal-Wallis one-way ANOVA test (*P* < 0.05) through the R stats package (R Core Team, 2020).

## Results

### Astronaut Microbiome Profiles

**FIGURE 2:**
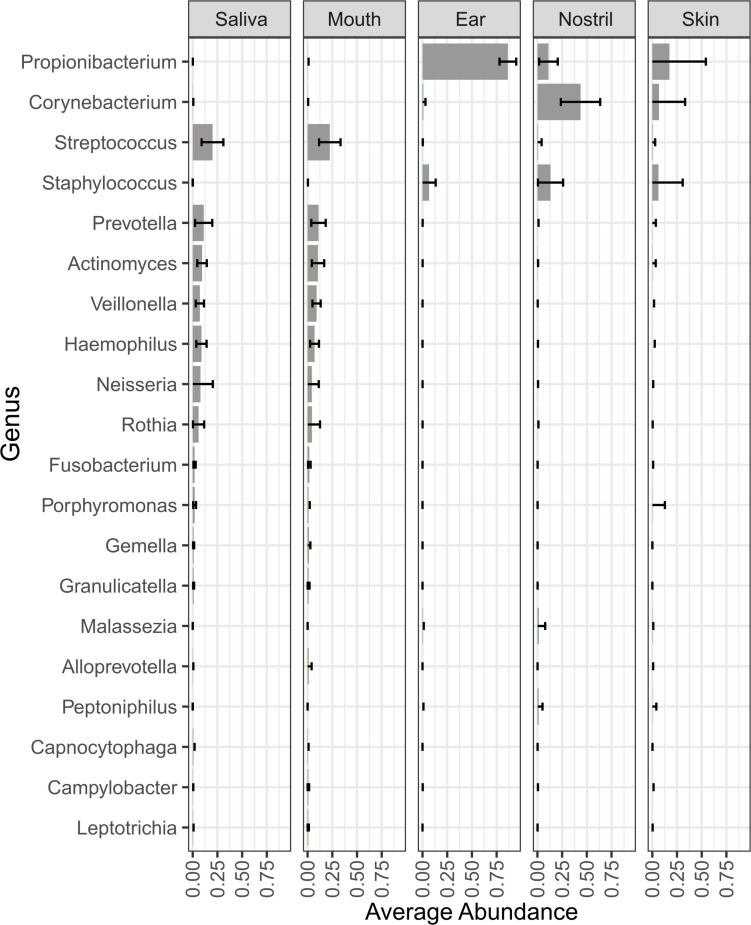
Genera with an average relative abundance of at least 1% across all body locations from all four astronauts. The bars in each column represent the average relative abundance of that genus across all samples from that body location.

For this study, 241 astronaut samples and 44 control samples were collected for a total of 285 samples. The samples were collected from four astronauts at seven time points from five body locations. For the saliva samples, four samples were collected at each time point at 2-day intervals. The average number of reads, the distribution in the number of reads per sample, and the percentage of reads belonging to bacterial, fungal, viral, and human sources are shown by sample type in Supplementary Table 1. The number of mapped reads and the LMAT taxonomic classification results for all control and astronaut samples are shown in Supplementary Data Sheet 1, and the relative abundance of species in each control samples is shown in Supplementary Figure 1. LMAT taxonomic classification for the astronaut samples identified 1,605 genera and 6,857 species across all 239 samples. Microbial genus (Figure 2) and species (Supplementary Figure 2) with an average abundance of at least 1% are shown, and the top five genus and species for each location are shown in Supplementary Tables 2, 3, respectively. The astronaut ear samples were dominated by *Propionibacterium* which had the highest average relative abundance across all body locations at 86.4%. *Propionibacterium* was also among the top five most abundant genera in the skin and nostril samples with an average relative abundance of 17 and 11%, respectively. The top three most abundant genera were the same for the ear, nostril, and skin samples (Supplementary Table 2). The top three most abundant genera in the skin samples (*Propionibacterium*, *Corynebacterium*, and *Staphylococcus*) are commonly isolated from the skin. The saliva and mouth samples were dominated by taxa associated with the oral microbiome (Supplementary Table 2). *Streptococcus, Prevotella, Actinomyces*, and *Haemophilus* were among the top five most abundant genera in the saliva and mouth samples with *Neisseria* and *Veillonella* rounding out the top five genera, respectively.

At the species level, the saliva and mouth samples were again very similar with *Haemophilus parainfluenzae*, *Rothia mucilaginosa*, *Actinomyces* sp. ICM47, and *Veillonella* sp. oral taxon 158 being in the top five most abundant species (Supplementary Table 3). *Staphylococcus epidermidis* was on average the most abundant species in the ear and nostril samples and the second most abundant species in the skin samples (Supplementary Figure 2). *Malassezia restricta* was the only other microbial species that was among the top five most abundant species in the ear, nostril, and skin samples (Supplementary Table 3). *Propionibacterium acnes* was in top five species for both the ear and skin samples, and *Propionibacterium* sp. KPL1844 and *Propionibacterium granulosum* completed the top five most abundant species in the ear samples. Several *Corynebacterium* spp. in the nostril and skin samples were among the five most abundance species for those body locations.

**FIGURE 3:**
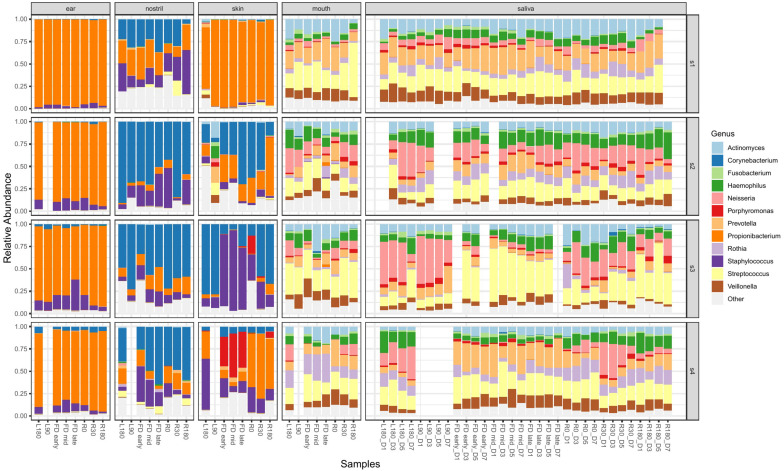
The top 12 genera with the highest relative abundance across all astronaut samples pre-, during and post-flight. The body locations are separated into individual columns and each astronaut is represented as a row. The samples in each cell are arranged in chronological order to display the change in the microbiomes over time.

Samples were collected from astronauts at multiple time points prior to, during, and post flight to identify changes in microbiome over time. Figure 3 shows the change in relative abundance of the top 12 species in ear, nose, skin, mouth, and saliva samples from all four astronauts collected pre-, during and post-flight. All 12 species are commonly associated with the human microbiome.

### Changes in Taxa Richness

The alpha diversity of each sample was measured according to the number of observed taxa (richness) and relative abundance of each taxon (Shannon index and Simpson index). The observed species richness for the LMAT (Supplementary Figure 3A) and Axiom Microbiome Array (Supplementary Figure 3B) results are shown. Due to the limited number of swab samples at an individual level, statistically significant changes between collection points were calculated using all four astronauts. No significant differences in the observed richness, exponentiated Shannon index, and reciprocal Simpson index were found between the eight collection time points.

**FIGURE 4:**
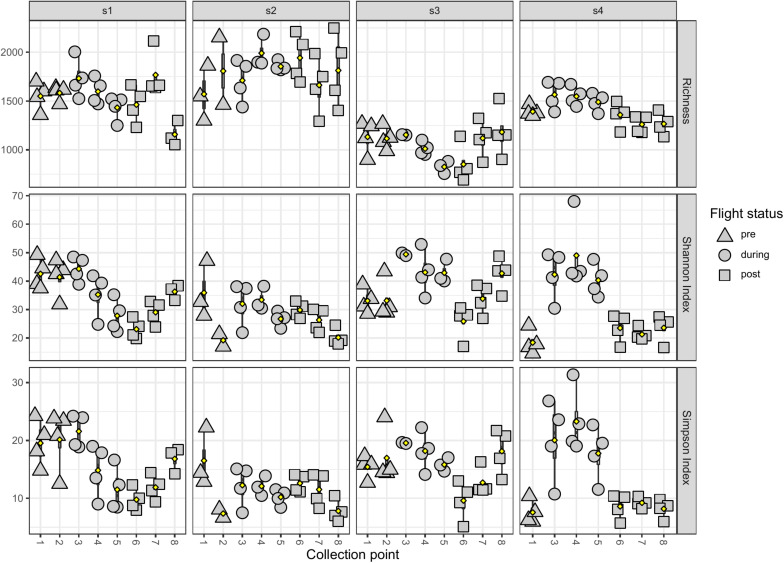
The observed species richness and evenness of astronaut saliva samples. The species richness (top row), Shannon index (middle row), and Simpson index (bottom row) are displayed by astronaut (s1 in the first column, s2 in the second column, s3 in the third column, and s4 in the fourth column) and samples are grouped by collection timepoints. The flight status of each sample is indicated by shape (pre-flight are triangles, during-flight are circles, and post-flight are squares).

LMAT alpha diversity changes in the saliva samples of each individual astronaut were also analyzed using the Kruskal-Wallis test (Figure 4). Samples were first grouped by flight status (i.e., pre-, during-, or post-flight). Astronaut 2 was the only member that showed no significant changes in the three alpha diversity metrics measured. Astronaut 1, 3, and 4 all showed significant changes (*P* < 0.05) in the Shannon and Simpson indexes. In Astronaut 1, the post-flight Shannon index (*P* = 0.00128) and Simpson index (*P* = 0.00225) were significantly different from the pre-flight levels. The during-flight Shannon index values for Astronaut 3 were significantly different from the pre-flight (*P* = 2.855e-3) and post-flight (*P* = 8.552e-3) Shannon index values. The Astronaut 3 during-flight Simpson index values was significantly different from the post-flight Simpson index values (*P* = 0.02296). Astronaut 4 had a significant increase in the Shannon (*P* = 3.208e-5) and Simpson index (*P* = 4.311e-5) during spaceflight. Astronaut 4 also displayed a significant difference in species richness between flight status groups. *Ad hoc* analysis revealed that the during-flight species richness was significantly different from the pre-flight (*P* = 0.01796) and post-flight (*P* = 1.774e-4) observed species richness.

### Changes in Oral Taxa Abundance During Flight

**FIGURE 5:**
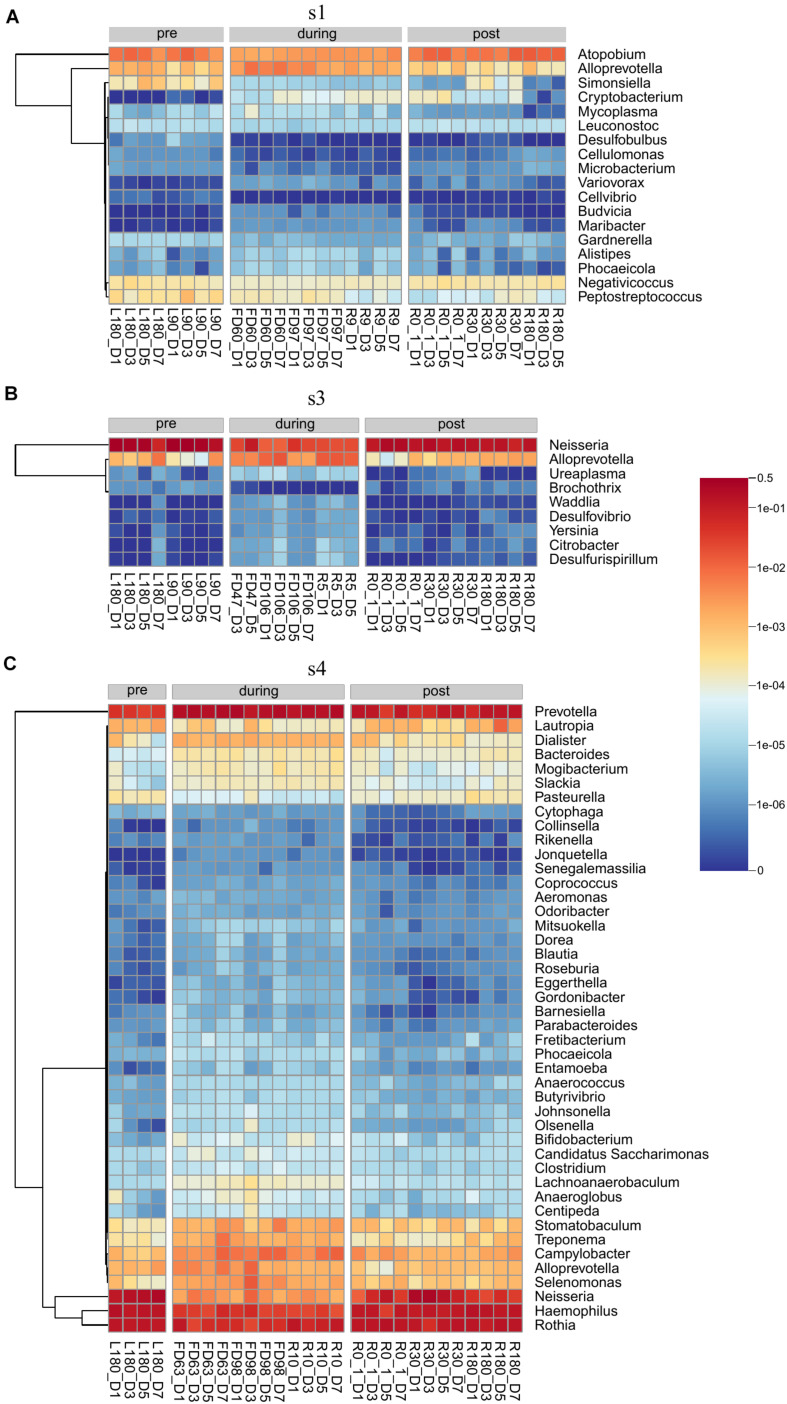
Heatmaps showing the relative abundance of genera identified as differentially abundant in Astronaut 1 **(A)**, Astronaut 3 **(B)**, and Astronaut 4 **(C)**.

Differential abundance analysis of the saliva samples was performed on each individual astronaut. Samples were grouped by flight status. Our analysis found a total of 67 genera (Supplementary Table 4 and Figure 5) and 206 species (Supplementary Table 5 and Supplementary Figures 4–6) that were differentially abundant. Astronaut 2 had no differentially abundant taxa. Among the remaining three astronauts, Astronaut 4 had the highest number of differentially abundant taxa with 44 genera (Figure 5C) and 173 species (Supplementary Figure 6). Astronaut 1 had the second most differentially abundant taxa with 18 genera (Figure 5A) and 35 species (Supplementary Figure 4), and Astronaut 3 had the third with 9 genera (Figure 5B) and 7 species (Supplementary Figure 5). *Alloprevotella* was the only genus that was differentially abundant in three astronauts, and the relative abundance of *Alloprevotella* increased during spaceflight and returned to pre-flight levels upon returning to Earth in all three astronauts (Figure 5). Two genera, *Neisseria* and *Phocaeicola*, were differentially abundant in two astronauts (Supplementary Table 4). *Neisseria* showed a significant decrease in relative abundance during spaceflight in Astronauts 3 and 4, and *Phocaeicola* showed a significant increase in relative abundance during spaceflight the Astronauts 1 and 4 (Figures 5A,C). In both cases, the relative abundance of *Neisseria* and *Phocaeicola* returned to pre-flight levels upon returning to Earth. The remaining 64 genera were only identified as differentially abundant in one astronaut.

No species were differentially abundant in three astronauts, but seven species were differentially abundant in at least two astronauts (Supplementary Table 5). *Alloprevotella rava, Alloprevotella tannerae, Megasphaera* sp. UPII 135-E, *Phocaeicola abscessus*, and *Prevotella* sp. oral taxon 473 all showed significant increases in their relative abundance during spaceflight in Astronauts 1 and 4. *Bacteroides massiliensis* showed a significant increase in relative abundance during spaceflight during Astronauts 3 and 4. Finally, *Actinomyces* sp. ph3 showed a significant decrease in relative abundance during spaceflight in Astronaut 1, but it showed a significant increase in relative abundance during spaceflight in Astronaut 4. *Actinomyces* sp. ph3 was the only species to display a significant change in relative abundance in multiple astronauts that were not consistent across the astronauts. The remaining 199 species were only identified as differentially abundant in one astronaut.

### Changes in Antimicrobial Resistance Markers

ShortBRED analysis of raw metagenomic reads from saliva samples identified 888 AMR markers. Kruskal-Wallis analysis of the saliva samples by flight status identified four AMR markers that were significantly different in all four astronauts (Supplementary Table 6). These included two erythromycin resistant markers (ARO:3000596 and ARO:3000498), a tetracycline resistant marker (ARO:3000168, and a *Streptomyces cinnamoneus* EF-Tu mutation (ARO:3003359). The tetracycline marker averaged less than 20 reads per collection point and the two erythromycin markers averaged less than 100 reads per collection points for all astronauts, so these markers were not investigated further. The read counts for the *Streptomyces* EF-Tu mutation marker (ARO:3003359) displayed a significant increase (*P* < 1.507e-5) in reads for the ARO:3003359 marker in all four astronauts’ post-flight samples. Astronauts 3 and 4 also had a significant decrease (*P* < 0.0002) in ARO:3003359 reads during flight, but this significant decrease was not observed in astronauts 1 and 2.

### Evaluating Sample Clustering Between Astronauts

**FIGURE 6:**
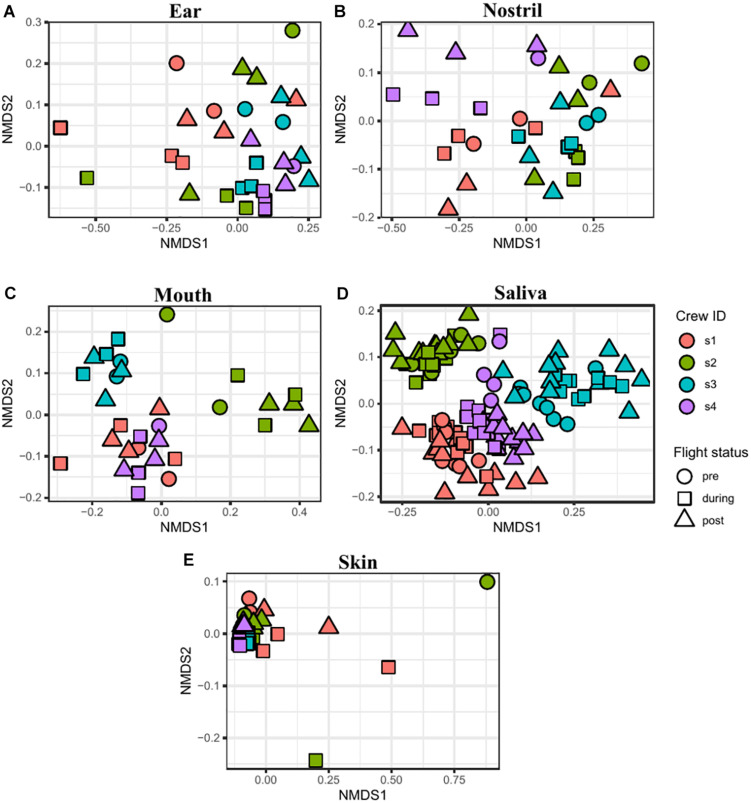
NMDS plots depicting the Jaccard distance between samples. Ear **(A)**, nostril **(B)**, mouth **(C)**, saliva **(D)**, and skin **(E)** samples are shown grouped by astronaut (Astronaut 1 or s1, red; s2, green; s3, blue; s4, purple) and flight status (pre, circle; during, square; post, triangle).

**FIGURE 7:**
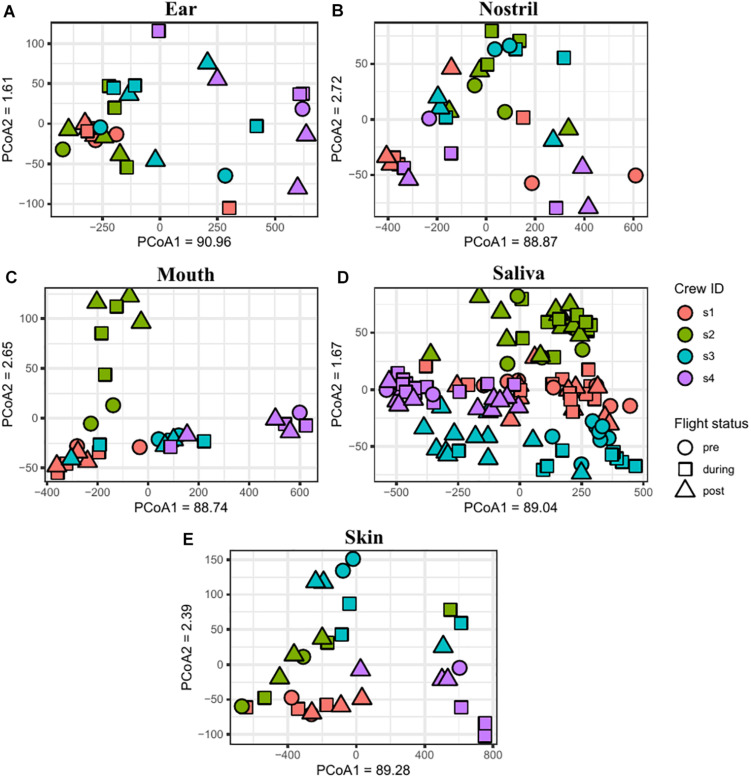
PCoA plots depicting the Euclidean distance between samples. Ear **(A)**, nostril **(B)**, mouth **(C)**, saliva **(D)**, and skin **(E)** samples are shown grouping by astronaut (s1, red; s2, green; s3, blue; s4, purple) and flight status (pre, circle; during, square; post triangle).

The similarity between samples was calculated using the Jaccard and Euclidean dissimilarities, and the resulting dissimilarity matrices were visualized using NMDS (Supplementary Figure 7A) and PCoA (Supplementary Figure 7B) plots, respectively. In both the NMDS and PCoA plots, the samples clustered into two separate groups with saliva and mouth samples clustered together and the skin, nostril, and ear samples clustered into a second group. To better visualize the associations between samples collected from the sample body location, NMDS (Figure 6) and PCoA (Figure 7) plots were generated for each body location. A PERMANOVA test was used to determine whether the samples grouped by either astronaut or flight status. PERMANOVA of the Jaccard dissimilarity showed that all body locations grouped by astronaut (*P* < 0.001), but not all body locations grouped by flight status. The ear (*P* < 0.001), skin (*P* = 0.008), and saliva (*P* < 0.001) samples are grouped by flight status, but the nostril (*P* = 0.095) and mouth (*P* = 0.079) samples did not meet our cut-off of *P* < 0.05. PERMANOVA of the Euclidean dissimilarity showed that the samples clustered by astronaut (*P* < 0.001) for all body locations except the nostril samples (*P* = 0.578). The saliva samples were the only body location that clustered by flight status (*P* = 0.011).

### Microarray Analysis of all Samples

The AMA detected 537 microbial targets across all astronaut samples. These targets represent 347 species consisting of 455 strains, 57 plasmids and 25 bacteria phages. All species detected by the AMA were also detected using metagenomics sequencing and LMAT. AMA detected species were checked against the Virulence Factor Database (VFDB) to identify opportunistic pathogens. Eleven opportunistic pathogens were detected across all 5 body locations (Supplementary Table 7). The prevalence of each species was calculated pre-, during-, and post-flight by body location. *Haemophilus influenzae* and *Streptococcus pneumoniae*, in the saliva samples, were the only two species to show a significant change in prevalence pre-, during, and post-flight. *H. influenzae* prevalence increased during spaceflight and was detected in 94% of samples. The prevalence of *H. influenzae* then decreased post-flight to 52% which was slightly higher than the pre-flight prevalence of 38%. *S. pneumoniae* showed an opposite trend with the prevalence dropping from 65% pre-flight to 38% during flight. The prevalence of *S. pneumoniae* decreased further post-flight to 25%.

## Discussion

Microbial pathogens in the ISS environment could pose a potential health risk for astronauts. Pre-flight testing and periodical in-flight monitoring have been performed for decades using culture-based analyses. The development of high-throughput sequencing technologies and subsequent research into the human microbiome has revealed the important role these microbial communities play in maintaining human health. Previous astronaut microbiome studies were limited to 16S rRNA based sequencing, which did not provide high resolution analysis at the genomic level (Voorhies et al., 2019), or only limited to one body location, which did not provide a broader view of the microbiome changes in different parts of the body (Garrett-Bakelman et al., 2019). In this study, the microbiome of four ISS astronauts was monitored prior to, during, and after their spaceflight missions using both a comprehensive microbial detection array as well as shotgun metagenomic sequencing. The microarray contains probes to detect more than 12,000 microbial species including viruses, bacteria, fungi, protozoa, and archaea (Thissen et al., 2019). The goal of the study was to characterize astronaut’s microbiome changes due to spaceflight conditions and identify any changes that may pose health risks to the astronauts during their mission.

This study monitored body locations with varying degrees of interaction with external environments. The microbiomes of the ear, nostril, and skin samples were dominated by skin associated bacteria (Avila-Herrera et al., 2020). Skin is the largest organ of the human body and the primary interface with the external environment. Skin serves as a physical barrier to prevent invasion of pathogens and education of the immune system (Byrd et al., 2018). ISS is a specialized enclosed built environment with continuous human inhabitants. The microbes in the ISS environment could impact the astronaut skin microbiome and vice versa. Avila-Herrera et al. reported potential bacterial exchanges between astronaut skin and ISS surfaces using metagenomic sequencing data from one astronaut and eight ISS surface locations from two consecutive flights (Avila-Herrera et al., 2020). Voorhies et al. also reported similarities between astronaut skin vs. ISS surfaces based on 16S sequencing (Voorhies et al., 2019). However, the results here did not show the four astronauts’ skin microbiomes shifting toward a consistent “spaceflight” microbiome while they were on board the ISS. Using the Jaccard distance, which measured differences in the species presence/absence profiles, the ear samples were the only body location that grouped by flight status, while all body locations grouped by astronaut. In addition, the Euclidean distance, which measured differences in the species’ relative abundance profiles, only showed samples significantly clustering by astronaut. The astronauts on board the ISS inhabit the spacecraft with two to five other astronauts. One astronaut will therefore be exchanging microbes with the ISS surfaces and their fellow crew; therefore, any microbiome shift will be different for each ISS crew. One exception to this could be the skin around the astronaut’s ear. The skin around the ear does not usually come into direct contact with a lot of other surfaces. The significant grouping of ear samples by their species profile in this study could be due to a spaceflight shift appearing in a body location where other external factors do not have a large influence. Further investigation of microbiome changes across entire ISS crews in both high and low contact locations will shed more light on the possibility of microbiome shifts in space.

Unlike the skin, the oral cavity is not in direct contact with the ISS surfaces, and the four astronauts maintained certain pre-flight oral microbiomes with unique species and abundances compared to the other astronauts. The oral microbiome is the second most diverse microbiome community in the human body (Deo and Deshmukh, 2019), and the composition of this community depends on physical state of a human, and a variety of external features including geographic region, diet, and oral health. Numerous studies have shown that diet affects the gut microbiome (Leeming et al., 2019), but very few studies are available regarding associations between diet and the oral microbiome (Lu et al., 2019). In one study, the abundance of *Neisseria* and *Haemophilus* is different between hunter-gatherers and westerners, and traditional farmers fall in between (Lassalle et al., 2018). Onboard the ISS, pre-cooked and packaged meals ensure most astronauts are exposed to similar diet and nutrition, so the differences of microbiome between different astronauts may be due to their individual microbiomes than diet and nutrition. Future microbiome studies of astronauts on different diets during spaceflight will shed light on the diet effects on the oral microbiome.

When averaging samples across of four astronauts, there was no significant change in alpha diversity between sample collection points for all five body locations. A previous publication on the saliva microbiome of astronauts reported changes at the individual level but not at the population level (Urbaniak et al., 2020) and such results were also supported by this study. An early analysis report of the Astronaut 1 saliva samples reported a decrease in alpha diversity during spaceflight (Avila-Herrera et al., 2020), but this trend was not consistent across all four astronauts. In this follow-up study, Astronauts 1, 3, and 4 all displayed significant changes in the Shannon and Simpson indexes. In Astronaut 1, the post-flight Shannon and Simpson indexes were significantly lower than the pre-flight indexes. This is due to a gradual decrease in the Shannon and Simpson indexes during spaceflight. Upon returning to Earth, these indexes were significantly lower than their pre-flight levels despite a gradual increase over time. In both Astronauts 3 and 4, the during-flight Shannon index was significantly higher than the pre-flight and post-flight levels. In all three astronauts, the changes in microbial diversity were not permanent and returned to pre-flight levels after returning to Earth.

The Shannon and Simpson indexes are measurements of the species evenness in the samples. Significant changes in these two metrics would be the results of changes in the relative abundance of species in the samples. Most genera and species identified as differentially abundant display increased relative abundance during-flight compared to pre-flight and post-flight. A general increase in the relative abundance of species would also increase the microbial evenness.

*Alloprevotella* significantly increased in Astronauts 1, 3, and 4. This genus (Downes et al., 2013) is closely related to the genus *Prevotella*, which also displayed significant increases in Astronaut 4 during-flight. *Prevotella* consists of several common oral species and increased *Prevotella* abundance has been linked to a diseased periodontal state (Abusleme et al., 2013). It is not possible to accurately predict the onset of oral disease by microbial content alone due to the variation of individual’s microbiome composition, but there is evidence that some taxa shift between healthy vs. diseased states (Schwarzberg et al., 2014). Further investigation into the temporal shift of the astronaut oral microbiome is needed to shed light on which taxa are regularly increases during-flight and if these are commonly associated with a healthy or infected oral cavity.

Antibiotics are commonly used in the treatment of periodontal disease (Schwarzberg et al., 2014). Our analysis found that four CARD AMR gene markers displayed significant changes in the number of reads in all four astronauts. The *Streptomyces* EF-Tu mutation (ARO:3003359) marker showed a significant increase in the number of reads post-flight in all four astronauts). This marker represents sequence variants of *Streptomyces cinnamoneus* elongation factor Tu that confer resistance to elfamycin antibiotics. The increase of EF-Tu mutations post-flight suggests that spaceflight conditions could have resulted in increased elfamycin resistance, and alternate antibiotics should be considered for any treatment post spaceflights.

In addition to metagenomic sequencing of the astronaut samples, the AMA was also used to detect the presence of microbial species from the astronaut samples. AMA is a faster and cheaper alternative to metagenomic sequencing. It has a semi-automated workflow and user-friendly software, but it does not provide quantitative read numbers like sequencing. The AMA contained 1.38 million probes that were designed to detect specific regions of target genomes. The detection of AMA uses stringent criteria in which more than 20% of the designed probes or greater than 8 probes need to be detected for a positive identification. In comparison, metagenomic sequencing uses >1 read as a positive detection. Therefore, the microarray detection may have a lower sensitivity than metagenomic sequencing. While AMA detected fewer species than LMAT, the detected taxa and changes in diversity were similar between the AMA and LMAT.

In summary, we evaluated how spaceflight affected the microbial composition of five locations across the human body. The results showed that the microbiome experienced a change in composition during spaceflight and returned to normal after the astronaut returns to Earth. Some astronauts, such as Astronauts 3 and 4, showed significant increases in taxonomic abundance and diversity, while Astronaut 2 showed very few changes. Analysis of the saliva samples revealed significant changes in some of the most abundant genera including *Neisseria* and *Prevotella*. Additional analysis of the saliva samples also found several AMR markers whose abundance significantly changed in all astronauts during their sampling period. However, despite the significant changes of these markers in all four astronauts, the markers did not display any constant trends across all four astronauts. These inconstant changes are probably the result of additional external forces such as the astronaut’s diet and microbial exchange with the ISS surfaces and the additional astronauts on board. In the future, it would be useful to monitor the microbiomes of the entire astronaut crew during an ISS mission. Tracking the entire crew could help identify the changes that are introduced from crew interactions, which will enable better characterization of the microbiome variations due to the environmental stresses of spaceflight.

## Data Availability Statement

The microbial sequencing associate with astronaut samples cannot be shared publicly due to IRB. The Data is available upon request in NASA Life Sciences Data Archive (LSDA) (https://lsda.jsc.nasa.gov/Dataset).

## Ethics Statement

The studies involving human participants were reviewed and approved by the Johnson Space Center Institutional Review Board. The patients/participants provided their written informed consent to participate in this study. Written informed consent was obtained from the individual(s) for the publication of any potentially identifiable images or data included in this article.

## Author Contributions

MM analyzed and interpreted the metagenomic and microarray data and was a major contributor in wrote the manuscript. JT performed sample processing, microarray and sequencing experiments, and helped with data curation. FK helped with project implementation, metadata, and administration. SM helped with project conceptualization, funding acquisition, and astronaut sample coordination. CU helped with sample processing, nucleic acid extraction, and sample and DNA archiving. KV helped with project conceptualization and funding acquisition. DS helped with project conceptualization and investigation. CJ helped with project conceptualization and funding acquisition, assisted with project administration, supervised the project, and contributed to the manuscript. All authors read and approved the final manuscript.

## Conflict of Interest

The authors declare that the research was conducted in the absence of any commercial or financial relationships that could be construed as a potential conflict of interest.
