# Supplementary material for: Investigation of Spaceflight Induced Changes to Astronaut Microbiomes
Source: Front Microbiol. 2021 Jun 2;12:659179. doi: 10.3389/fmicb.2021.659179 (PMC8207296; doi:10.3389/fmicb.2021.659179)
Supplement: Supplementary file 8 [file Data_Sheet_1.PDF]

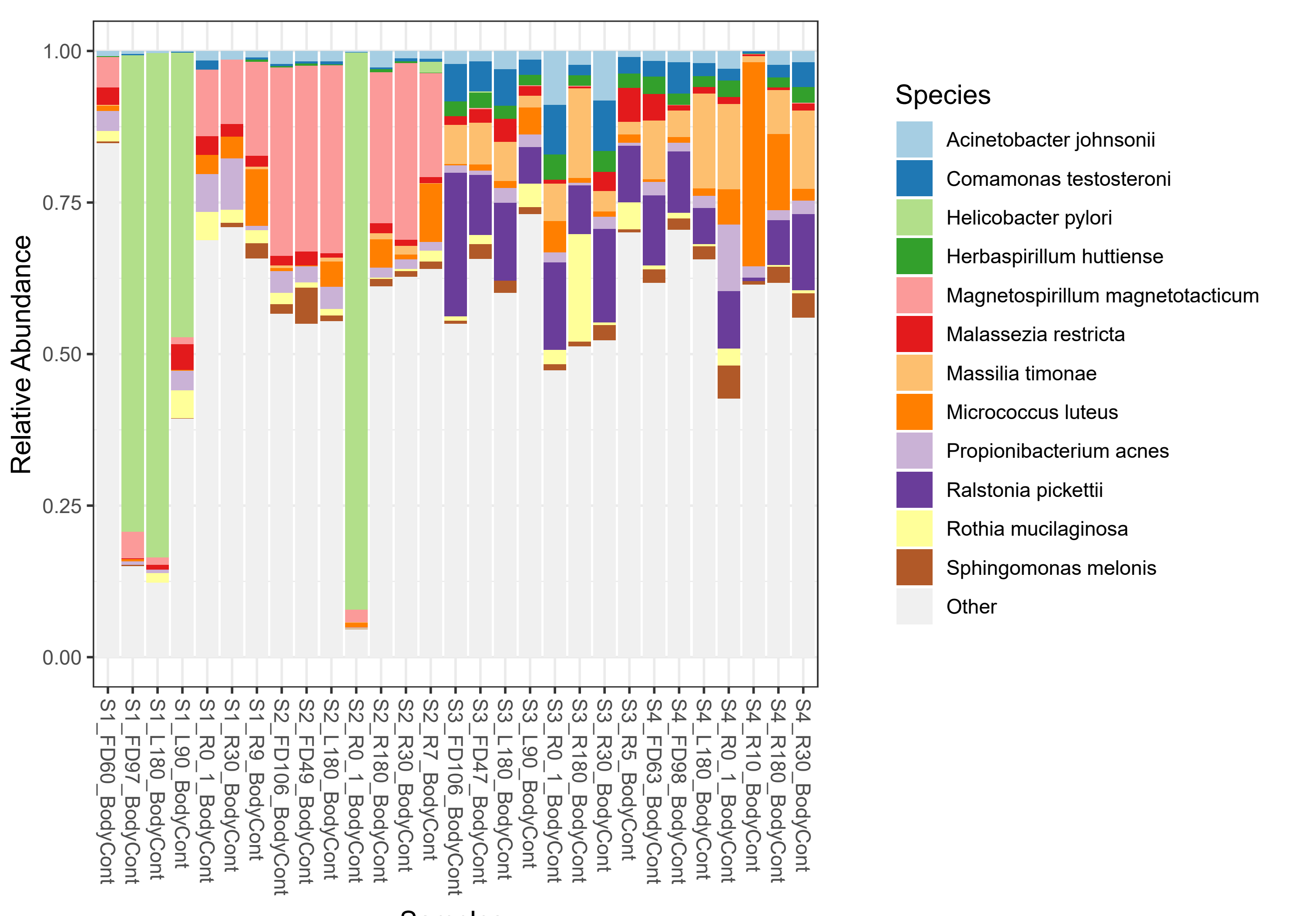

Figure S1. The top 12 species with the highest relative abundance across all control samples.

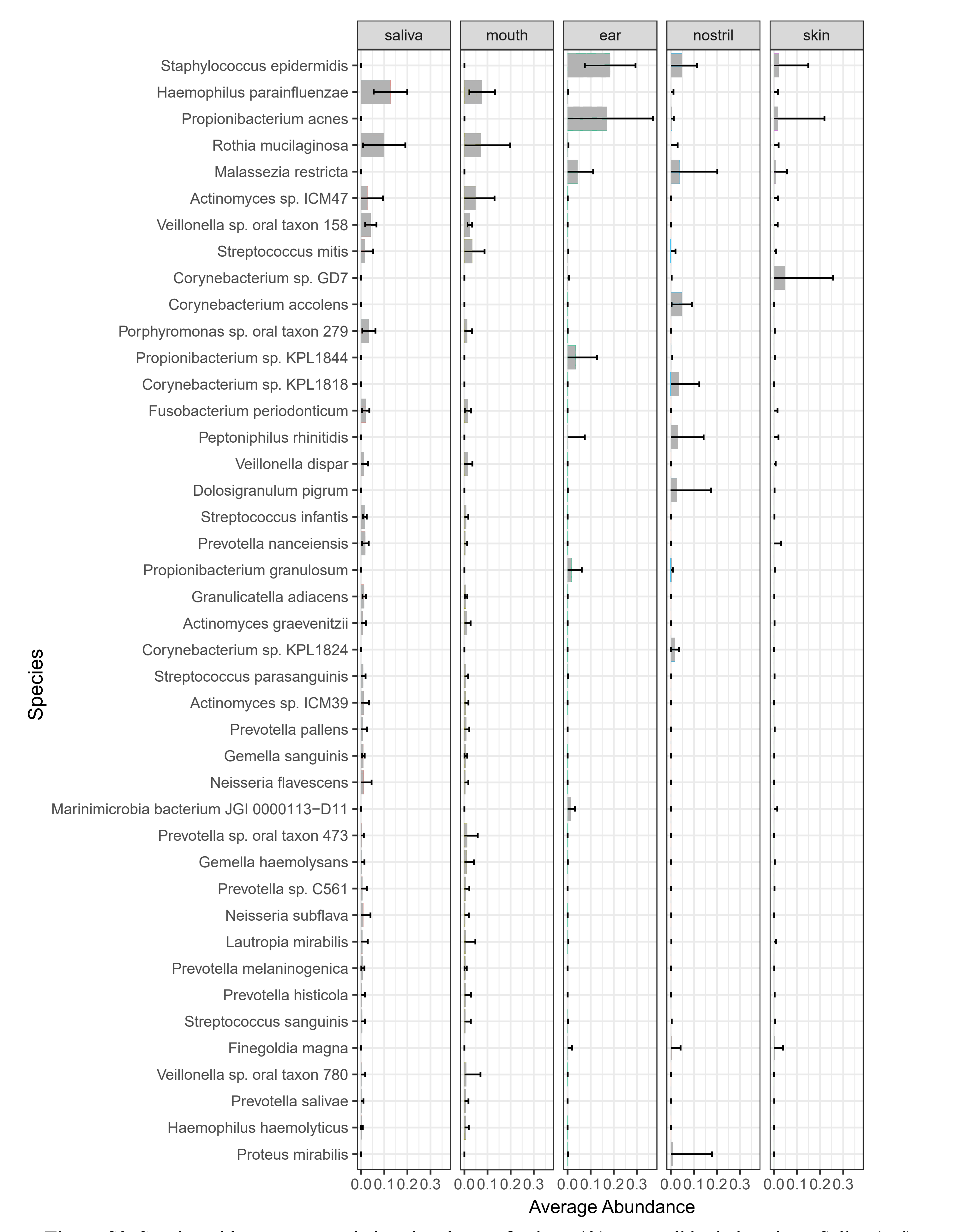

Figure S2. Species with an average relative abundance of at least 1% across all body locations. Saliva (red), mouth (yellow), ear (green), nostril (blue), and skin (purple) results are seperated into columns. The bars in each column represent the average relative abundance of that species across all samples from that body location.

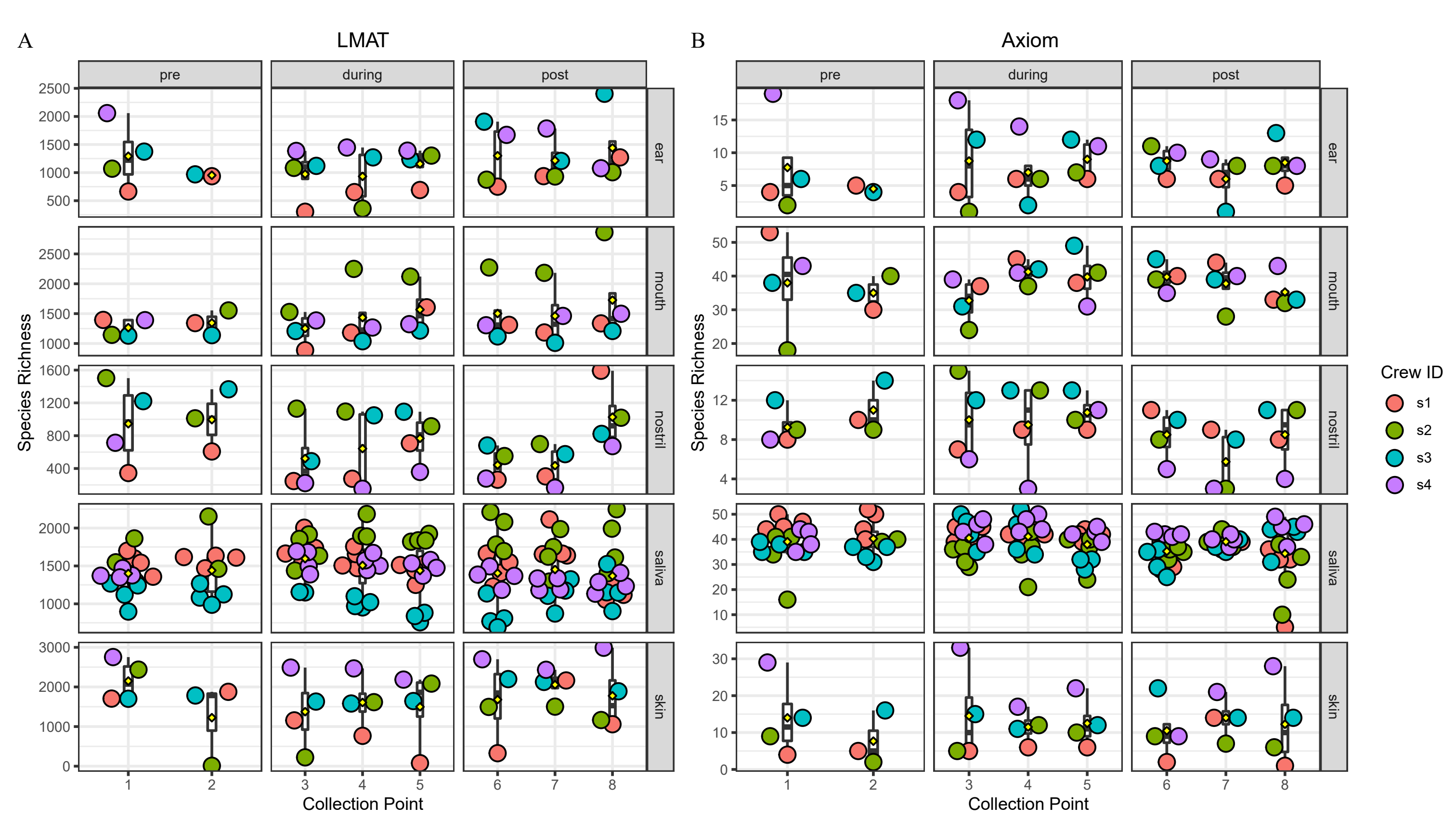

Figure S3. Obsereved species richness in astronaut samples. LMAT (A) and Axiom Microbiome Array (B) results are seperated by flight status (columns) and body locations (rows). Astronaut 1 (red), Astronaut 2 (green), Astronaut 3 (blue), and Astronaut 4 (purple) were grouped by collection point. A box plot shows the 1st, 2nd, 3rd, and 1.5 times the interquartile range of each time point's alpha diversity sample distribution. Yellow diamonds represent the means.

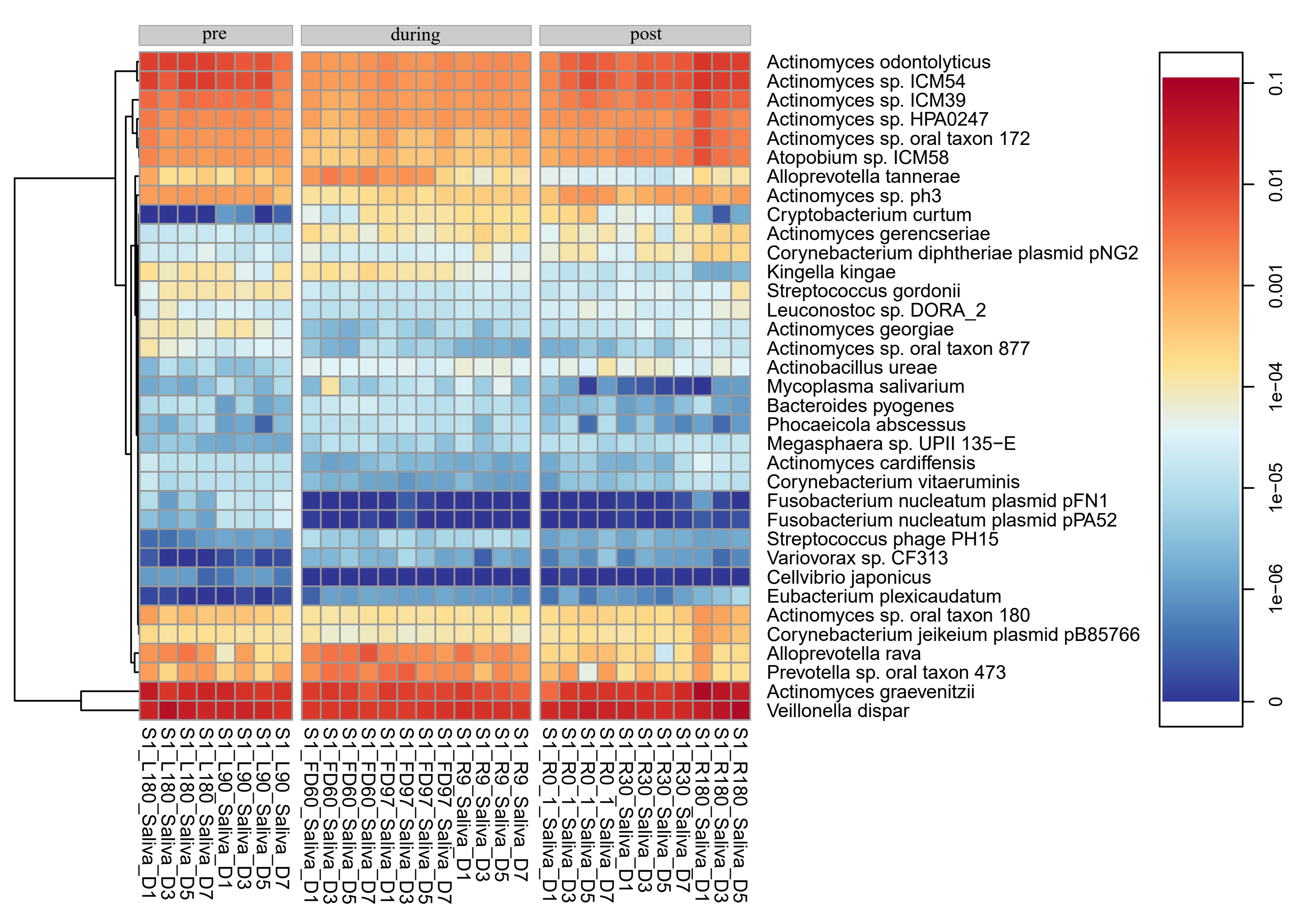

Figure S4. Heatmap depicting the relative abundance of species identified as differentially abundant in Astronaut 1 saliva samples. Samples were grouped by flight status (i.e. pre, during, and post flight) and species with P-values < 0.05 were considered significant. All P-values were adjusted using the Benjamini-Hochberg method.

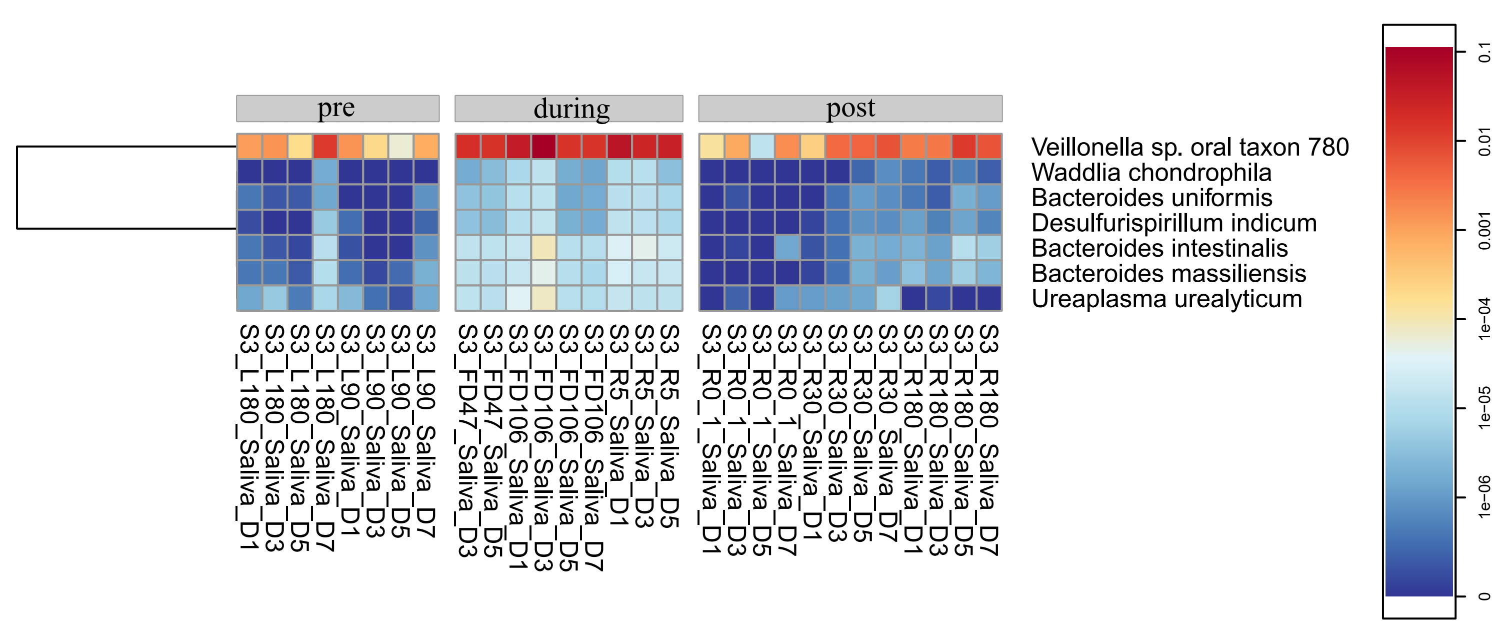

Figure S5. Heatmap depicting the relative abundance of species identified as differentially abundant in Astronaut 3 saliva samples. Samples were grouped by flight status (i.e. pre, during, and post flight) and species with P-values < 0.05 were considered significant. All P-values were adjusted using the Benjamini-Hochberg method.

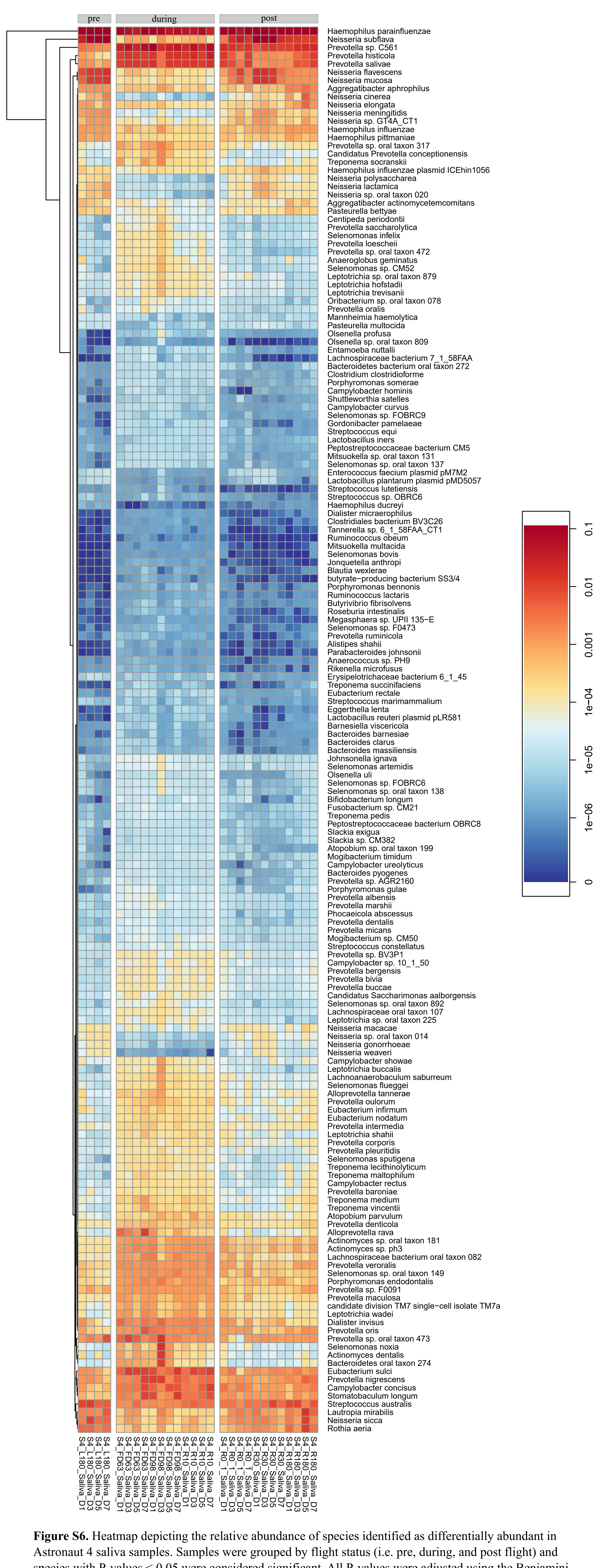

Figure S6. Heatmap depicting the relative abundance of species identified as differentially abundant in Astronaut 4 saliva samples. Samples were grouped by flight status (i.e. pre, during, and post flight) and species with P-values < 0.05 were considered significant. All P-values were adjusted using the Benjamini-Hochberg method.

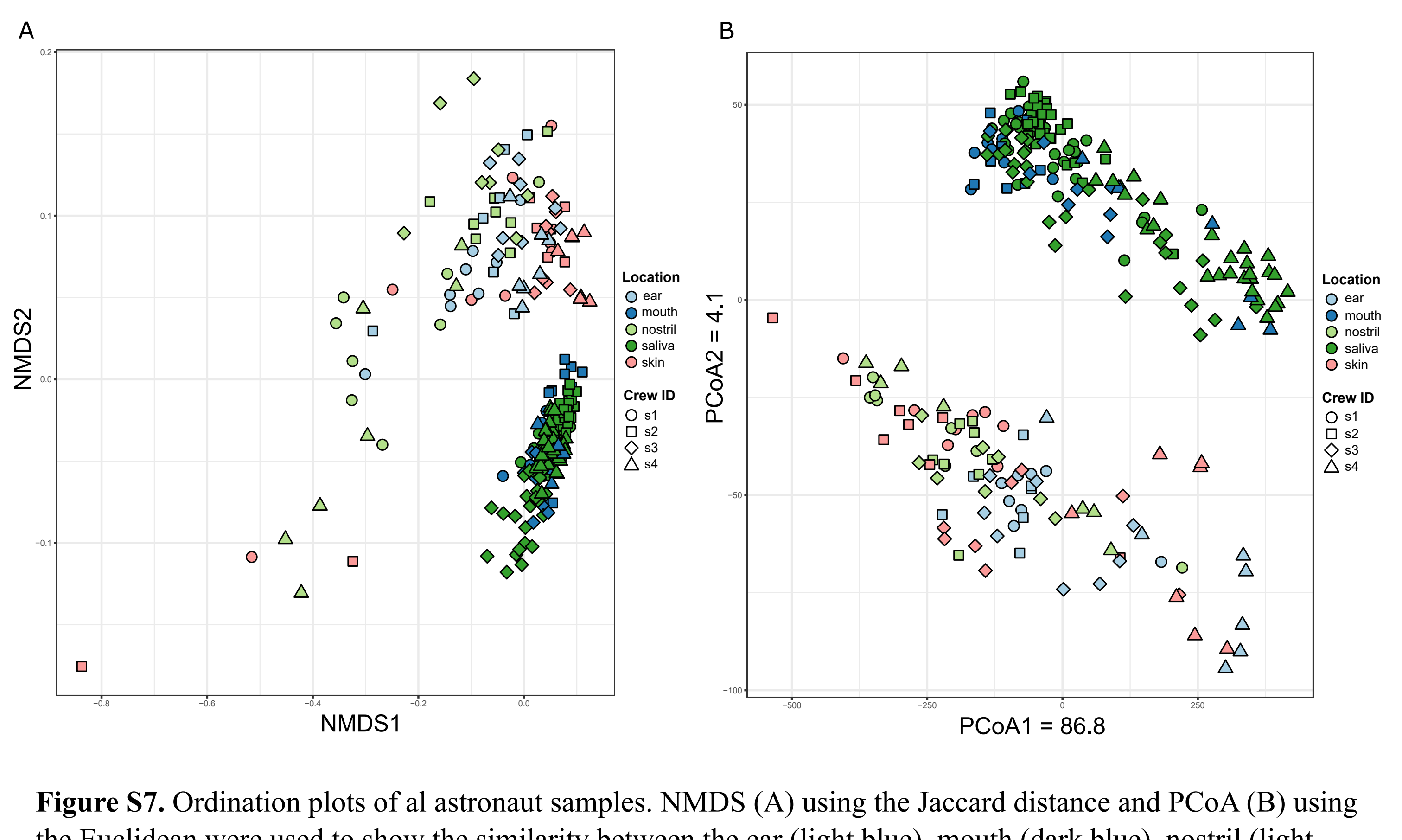

Figure S7. Ordination plots of al astronaut samples. NMDS (A) using the Jaccard distance and PCoA (B) using the Euclidean were used to show the similarity between the ear (light blue), mouth (dark blue), nostril (light green), and skin (red) samples.
